# A mixed methods study of suicide protective factors in college students

**DOI:** 10.1186/s41155-024-00315-0

**Published:** 2024-09-13

**Authors:** Hareli Fernanda Garcia Cecchin, Hellen Emily Rodrigues da Costa, Gabriela Ramos Pacheco, Gabriel Barcellos de Valencia, Sheila Giardini Murta

**Affiliations:** 1 PROEST, Universidade Federal do Tocantins, Quadra 109 Norte, Av. NS 15, ALCNO-14, Bloco da Reitoria, Térreo, Plano Diretor Norte, Palmas, TO CEP 77001-090 Brazil; 2 Instituto de Psicologia, Universidade de Brasília, Brasília-DF, Brazil

**Keywords:** Suicide prevention, Protective factors, College student, Mental health promotion, Mixed methods study, Socioecological model

## Abstract

**Introduction:**

Mental health professionals, teachers, families, and public administrators are concerned about suicide rates among young people, particularly in the university context. For every ten college students worldwide, three attempt suicide in their lifetime, and two experience suicidal ideation. Reducing these rates requires interventions that recognize the problem in terms of risk factors and protective factors.

**Objective:**

The general aim of the study was to map the protective factors for suicide among college students, as perceived by them, mental health professionals, and coordinators of undergraduate courses in a public university in the North of Brazil.

**Methods:**

The study followed an exploratory, mixed-method design. Data were collected through interviews and the application of a questionnaire with 54 participants, including college students (*n* = 20), mental health professionals (*n* = 22), and course coordinators (*n* = 12). Data were analyzed using Content Analysis and simple descriptive statistics.

**Results:**

The findings show that the protective factors for suicide most cited among the three groups were social support, strengthening of internal resources, institutional support, and finding meaning about the change to enter the university. Although the three audiences did not converge, the protective factors also frequently reported were psychological treatment, leisure activities, religious engagement, medical treatment, civic engagement, employability, opportunities for social ascension offered by the university, and quality family relationships.

**Conclusion:**

It is suggested that these protective factors are considered when formulating policies to promote mental health and suicide prevention in the university environment.

## Introduction

Mental health professionals, teachers, families, and public administrators are concerned about suicide rates among young people. A meta-analysis estimated that 22.3% of college students worldwide experience suicidal ideation, and 3.2% attempt suicide in their lifetime (Mortier et al., 2018). Some subgroups of this population are more likely to develop these problems, such as black youths (Ministério da Saúde, 2018), gender minorities (Di Giacomo et al., 2018), or those who combine two minority identities (Shadick et al., 2015), such as young black and homosexual men.

Reducing these rates requires interventions that reduce risk factors and strengthen protective factors. Various national and international studies have identified suicide protective factors for young people at different levels. At the individual level, the following have been identified as protective factors: self-esteem, self-efficacy, social skills (Pereira et al., 2018), a sense of hope, having reasons to live (Wenjing Li et al., 2020), and practicing sport (Aragão, 2019; Nomura et al., 2021). At the interpersonal level, quality family relationships, friendships (Pereira et al., 2018), and protective social networks have been found to be relevant (Ribeiro & Moreira, 2018). At the organizational level, university culture factors, such as support and inclusion, have also been identified (Venturini & Goulart, 2016).

Although the recognition of risk and protective factors is considered essential for the design and implementation of suicide prevention programs, literature reviews on suicide risk and suicide protective factors show that scientific production has focused more on risk factors than on protective factors (Franklin et al., 2017; Luong et al., 2018; Miranda-Mendizabal et al., 2019; Wenjing Li et al., 2020). Furthermore, these review studies demonstrate that the USA and other high-income countries prevail in researching suicide protective factors for college students. This limits generalization to other contexts, such as low- and middle-income countries. It is therefore crucial to examine protective factors in low- and middle-income countries, which have different social contexts.

Research that focuses on protective factors is vital to guide the planning of suicide prevention programs. Programs should not be exclusively designed to focus on risk reduction and should also include health promotion, considering the individual who has the problem and the physical and social environments that surround them, in a socioecological approach (Bartholomew-Eldredge et al., 2016; Kok et al., 2017; Peters, 2014). Notably, protective factors are not reverse risk factors and generally include cultivating socio-emotional skills (Suh & Jeong, 2021) and interpersonal and community resources.

Furthermore, this multilevel view requires a systemic, complex, and integrated approach. Although protective factors have been analyzed individually and in isolation by studies, they are cumulative and interdependent (Pereira et al., 2018). Social support, for example, can increase other protective factors, such as self-esteem, a sense of belonging and support to deal with stressful events, and symptoms of psychopathologies (Kleiman & Liu, 2013).

This approach has encouraged the use of multiple informants in research directed toward a more comprehensive understanding of the phenomenon. Connor and Rueter, (2009) assessed the risk of suicide in adolescents based on observation and interviews with mothers, fathers, and siblings, as well as adolescents with suicidal ideation. Wittink et al., (2006) examined physician–patient agreement and disagreement regarding the patients' depression status. Consulting multiple informants can help to balance the results, given that when data are collected through self-reports, consulting only one type of interviewee can bias the results. For Atkins et al., (2017), the individual who has the problem will likely perceive it as being related to external factors rather than to internal factors. In the study by Connor and Rueter, (2009), young people mentioned more individual factors, while parents and teachers focused on environmental factors. This research demonstrates that combining multiple informants allows for a more complete assessment of the problem, as recommended in the planning of health-promoting intervention approaches (Bartholomew-Eldredge et al., 2016).

Based on the above, the study’s general aim was to map the protective factors for suicide among young people as perceived by college students, mental health professionals, and coordinators of undergraduate courses in a public university in the Northern region of Brazil.

## Method

**Fig. 1:**
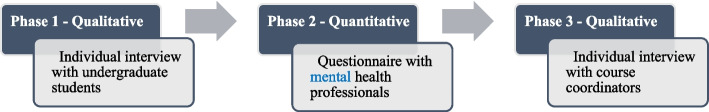
Visual template of the research strategy (adapted from Creswell, 2010)

The study design followed a mixed exploratory method, with a strategy of triangulating interviewees (Creswell, 2010; Morgan, 1998). The composition of the study was organized using the Mixed Methods Article Reporting Standards (MMARS) of the American Psychological Association–APA (Levitt et al., 2018). The methods were used in sequence to investigate protective factors for suicidal behavior, as suggested in the model by Steckler et al., (1992). The study used interviews with students and course coordinators and a questionnaire with mental health professionals in three data collection stages. Figure 1 summarizes the sequence and audience of the data collection.

### Context

This study was conducted at a public university in the North of Brazil that offers undergraduate and graduate courses in various areas of knowledge. The university is comprised of 5 campuses located in 5 municipalities. This institution offers monthly financial assistance to low-income students experiencing severe mental distress. The student must use the assistance for mental health treatment, which includes medical and/or psychological treatment and medication.

### Participants

The 54 study participants included undergraduate students, mental health professionals, and undergraduate course coordinators. A total of 20 undergraduate students who had suicidal ideation in 2020 participated. The students were selected after accessing the institutional database with information on students who received financial aid from the university to undergo mental health treatment in 2020. The original sample consisted of 118 students, 50 of whom had suicidal ideation reported by a health professional, with 20 selected for an interview. The number of participants was determined following Francis et al., (2010): a minimum of 10 interviews followed by three additional interviews until no new themes emerged. Accordingly, the minimum number of interviews suggested by the literature was doubled to ensure data saturation.

The selection of students with suicidal ideation was carried out to achieve a balanced division between the campuses and when the individual received the assistance, giving preference to those who had received assistance for a longer period, indicating it was a more serious case. This division was made by organizing the database in the following way: students with suicidal ideation were selected. Then, they were arranged from the longest to the shortest participation time, and subsequently separated into five lists, according to the campus the student was connected to. One campus did not have any students receiving the assistance in the year mentioned above. The first 20 students from each campus who responded to the contact were interviewed (see Table 1). The inclusion criteria for the students were receiving financial assistance from the university for health treatment (medical and/or psychological consultation and purchase of medication) in 2020, and having presented suicidal ideation at the beginning of the treatment. The students were of low socioeconomic status.

**Table 1:** Sociodemographic and educational data of the students

Name	Gender	Sexual orientation	Age	Ethnicity	Course	Course modality
Participant 1	Female	Heterosexual	23	Mixed race	Forest Engineering	Full time
Participant 2	Female	Heterosexual	23	Black	Bioprocess Engineering	Full time
Participant 3	Male	Heterosexual	24	Black	Letters	Morning
Participant 4	Female	Heterosexual	24	Mixed race	Psychology	Full time
Participant 5	Male	Heterosexual	28	White	Agronomy	Full time
Participant 6	Female	Homosexual	23	Mixed race	Psychology	Full time
Participant 7	Male	Homosexual	24	Mixed race	Pedagogy	Morning
Participant 8	Female	Heterosexual	27	Black	Social Services	Morning
Participant 9	Female	Homosexual	23	Black	Journalism	Morning
Participant 10	Female	Heterosexual	25	Mixed race	Environmental Engineering	Full time
Participant 11	Female	Homosexual	25	Indigenous	Medicine	Full time
Participant 12	Female	Homosexual	32	Black	Pedagogy	Morning
Participant 13	Female	Heterosexual	23	White	Forest Engineering	Full time
Participant 14	Female	Heterosexual	21	Mixed race	Bioprocess Engineering	Full time
Participant 15	Male	Heterosexual	21	Mixed race	Bioprocess Engineering	Full time
Participant 16	Female	Heterosexual	25	Mixed race	Agronomy	Full time
Participant 17	Female	Bisexual	25	Black	Medicine	Full time
Participant 18	Female	Heterosexual	25	Mixed race	Psychology	Full time
Participant 19	Female	Homosexual	23	Mixed race	Psychology	Full time
Participant 20	Female	Homosexual	24	Mixed race	Social Sciences	Morning

A total of 41 mental health professionals who assisted the students with mental health difficulties were invited to participate. These mental health professionals were community providers. Among the 41 mental health professionals, 22 (53.6%) agreed to participate in the study (Table 2). The mental health professionals assisted from 1 to 11 students (*M* = 1.63; SD = 2.06).

**Table 2:** Sociodemographic data of the mental health professionals

Variable	Description	Profession		Total
		Physician	Psychologist	
Gender	Female	2 (12%)	14 (87%)	16 (100%)
	Male	4 (66%)	2 (33%)	6 (100%)
Total	––––––––	6 (27%)	16 (73%)	22 (100%)

The institution had 37 undergraduate course coordinators and all of them were invited to participate. Course coordinators are the professors who have the most contact with administrative sectors, including the ones dealing with students’ mental health. Due to this direct and ongoing interaction, the course coordinators are essential in identifying students who need support and referring them to the appropriate services. Approximately one-third of the course coordinators (*n* = 12) accepted the invitation to participate (Table 3).

**Table 3:** Sociodemographic and educational data of the course coordinators

Name	Gender	Course	Education level	Working time
Participant 1	Male	Environmental Engineering	Doctorate	8
Participant 2	Male	Civil Engineering	Doctorate	8
Participant 3	Female	Law	Doctorate	7
Participant 4	Male	Physical Education	Master’s	0.5
Participant 5	Female	Forest Engineering	Doctorate	3
Participant 6	Female	Nutrition	Doctorate	8
Participant 7	Female	Social Services	Doctorate	10
Participant 8	Female	Law	Master’s	3
Participant 9	Female	Field Education	Master’s	4
Participant 10	Female	Journalism	Doctorate	17
Participant 11	Female	Pedagogy	Doctorate	8
Participant 12	Male	Administration	Doctorate	3

Working time is in years and refers to the length of time of working in the institution

### Instruments

The interviews and questionnaires used were developed based on research questions and the literature on needs assessment (Bartholomew-Eldredge et al., 2016; Kok et al., 2017). The interviews were conducted with students and course coordinators by a researcher familiar with the institution’s culture. Students were asked the following questions: “After you asked for help, what or who helped you to deal with these problems?” and “Of all the things that helped you deal with the suffering, which one(s) helped most?” The coordinators were asked the question: “How does university life affect the student's mental health?” If the respondent provided a response addressing negative issues, they were then asked, “Do you think university life positively affects student mental health?”.

The questionnaire was used with mental health professionals and contained the following question: “What protective factors have you observed in the university students you have assisted?” The response was analyzed considering the codes obtained after analyzing the student interviews. Nine factors from the literature were also considered: self-efficacy, high self-esteem, university culture factors (reception, inclusion, etc.), social skills, organization of the study routine, quality family relationships, sense of hope, having reasons to live, and medical treatment. The semi-open question allowed the professional to add other answers.

### Procedures

#### Data collection

The study had three data collection phases. Phase 1 consisted of interviewing students who had received assistance for mental health treatment. The students were recruited via email, phone call, and messaging, and asked to participate in a private videoconference interview, on a date and time chosen by the participant. The interviews lasted from 17 to 50 min, with a mean of 28 min. The videoconference interviews were later transcribed.

Phase 2 consisted of applying an online questionnaire with the mental health professionals who had assisted the students who received financial assistance for treating mental health difficulties. After the students were interviewed, the researchers returned to the database and verified the mental health professionals who assisted the 50 students with suicidal ideation in 2020. The email and telephone data of the mental health professionals were extracted from the students’ medical records. The database query returned the contacts of 41 mental health professionals who assisted the students. The mental health professionals were recruited via email, phone calls, and messaging. A link to the questionnaire was sent via email to all mental health professionals who assisted the students who received financial assistance in 2020.

In phase 3, the undergraduate course coordinators were individually interviewed. The undergraduate course coordinators were recruited through email invitation for an individual interview via videoconference, on a date and time to be chosen by them. The third phase was added because some organizational factors had been cited in the students’ interviews. Interviewing the course coordinators aimed to investigate whether these factors were also noticed by professors. Due to the vast number of professors at the institution, it was decided to interview only the course coordinators. All undergraduate course coordinators at the university were sent an email inviting them to participate in a research study that has significant implications for student support. All those who responded to the email (*n* = 12), confirming their interest in contributing to the study, were interviewed. The interviews were carried out through videoconference and lasted from 20 to 74 min, with a mean length of 46 min.

#### Data analysis

Data analysis was carried out in three phases. First, two researchers individually analyzed the data from each audience. The interviews with students and coordinators were transcribed and analyzed using Content Analysis (Bardin, 2011), and the mental health professionals’ responses to the questionnaire were analyzed using descriptive statistics. Next, data from each audience were individually categorized according to the 5 dimensions of the socioecological model (individual, interpersonal, organizational, community, and social). Categorizing the data within the socioecological model allowed it to capture the complexity of interactions between the different factors influencing students’ mental health. Finally, the three databases were analyzed separately and later grouped into large categories, inductively, according to the proximity of the themes, as shown in Table 4.

**Table 4:** Organization of protective factors according to the codes and categories constructed

Level	Category	Code
Individual	Psychological treatment	Psychotherapy
	Medical treatment	Drug therapy
	Complementary treatment	Essential oils
		Body therapy
		Complementary therapies (Bach flower remedies and Reiki)
	Leisure activities	Physical activity practice
		Drawing
		Taking walks with friends
		Living with a pet
	Temporary leave of absence from the university	Temporary leave of absence from the university
	Find meaning in life	Finding meaning for choosing to study at the university
		Opportunity for personal fulfillment, especially by identifying with the course
	Strengthening internal resources	Expanding autonomy
		Inserting oneself into a job or internship and organizing one’s study routine
		Learning to resolve conflicts
		Self-efficacy
		sense of hope
		high self-esteem
Interpersonal	Social support	Family Support
		Affective partner support
		Support from friends / Making new friends
	Living with people who have had a mental disorder	Interacting with people who have had a mental disorder
	Quality family relationship	Quality family relationship
Organizational	Institutional care	Support from teachers
		Support from administrative technicians
		University cultural factors, such as welcoming and inclusion
	University as a refuge from problems	University as an escape from the problems young people faced with their families
Community	Social engagement	Being part of a religion
Social	Civic engagement	Participating in social movements
	Employability and social opportunities offered by the university	University courses provide opportunities for expanding job opportunities, enabling better living conditions
		The COVID-19 pandemic enabled more flexible teaching and facilitated access to knowledge

The students’ interviews combined inductive and deductive thematic Content Analysis. The objective was to identify the codes in the participants’ responses and, subsequently, deductively validate these codes to those that appear in similar studies. Literature findings were considered, however, there was flexibility for creating new categories. Thematic analysis was also used in the interviews with the course coordinators, but only inductively because there were no similar studies on which to base the creation of categories.

The member-checking procedure, systematized by Birt et al., (2016), was used to evaluate the reliability of the qualitative data. A data summary was sent to students and course coordinators, and they were asked to read and comment on whether the results described their experience. The participants were warned that reading the text could cause discomfort and the main researcher’s contact was provided for use if deemed necessary. None of the participants requested adjustments or corrections in the results.

All participants were duly informed about the research process and consented to participate by signing a consent form. The study was approved by the Ethics Committee for Research in Human and Social Sciences of the University of Brasília under CAEE nº 42,405,021.3.0000.5540 and carried out following CNS Resolution number 510/2016.

## Results

Data analysis identified individual, interpersonal, organizational, community, and social protective factors, mentioned by college students, professionals, and undergraduate course coordinators. Considering the convergences between interviewees, the most frequently reported suicide protective factors were social support, strengthening of internal resources, institutional support, and meaning of life. Next, psychological treatment, leisure activities, religious engagement, medical treatment, and civic engagement were most frequently reported and consistent among students and mental health professionals. Although reported only by course coordinators or mental health professionals, employability and social mobility opportunities offered by the university, and quality family relationships were, respectively, cited fairly frequently. Other protective factors less frequently reported were complementary treatment, leaving the course and temporary leave of absence from the university, interacting with people who have had a mental disorder, and the university as a refuge from family conflicts. Table 5 shows these data. Next, the results will be presented according to each interviewee in the order of the frequency with which they were cited.

**Table 5:** Frequency of categories of protective factors according to students, mental health professionals, and undergraduate course coordinators

Level	Category	Students (*n* = 20)	MHP* (*n* = 22)	Coor* (*n* = 12)
Individual	Psychological treatment	80.0%	86.4%	0
	Medical treatment	10.0%	50.0%	0
	Complementary treatment	10.0%	9.1%	0
	Leisure activities	33.3%	81.8%	0
	Leaving from the course and temporary leave of absence from the university	5.0%	4.5%	0
	Meaning of life	5.0%	22.7%	25.0%
	Strengthening of internal resources	15.0%	81.8%	50.0%
Interpersonal	Social support	95.0%	90.9%	25.0%
	Interacting with people who have had a mental disorder	5.0%	13.6%	0
	Quality family relationship	0	36.4%	0
Organizational	Institutional welcome	20.0%	18.1%	25.0%
	University as a refuge for family conflicts	0	0	8.3%
Community	Religious engagement	25.0%	27.3%	0
Social	Civic engagement	10.0%	31.8%	0
	Employability and opportunities for social ascension	0	0	58.3%

*MHP Mental Health Professionals. *Coord = undergraduate course coordinators

### Protective factors for suicide as perceived by students

#### Social support

The most frequently reported category was social support, with 95% of students mentioning support from friends (60%), family (25%), and a romantic partner (20%) as a factor that helped them cope with their distress. The students often reported that therapy helped them make new friends or strengthen existing bonds.

“From the moment I started doing therapy, I started to create new relationships through the therapy. I started to make new friends through that. And I have those friendships to this day. And those friendships helped me a lot when I was at a low point. (...) We work together, and we always talk. So, I think friendship was a factor that really influenced my development. When we talked, she also commented “ah, you were also my first friend in this classroom”, because she was also sort of isolated from the students. So, it worked. Two outcasts getting together and forming a friendship: (Participant 3).

#### Psychological treatment

The second most frequently reported category was psychological treatment, with 80% of the participants mentioning this. The students’ narratives stated that psychotherapy helped them feel welcome, strengthened their self-esteem, reduced the pressure on themselves, perceived new possibilities for solving problems, gave new meaning to past traumas, and helped them deal better with the demands of adult life, and establish a healthier relationship with parents and intimate partners. They also reported that they overcame social anxiety, made new friends, felt more integrated with their classmates, and had less difficulty doing group work.

“After therapy I experienced a significant improvement. It’s like my therapist took a sculpture, in fact, she took clay and turned it into a sculpture. Therapy helped me create the resources for an adult life that I didn't have in the past. (...) And then there are self-esteem issues, several issues were addressed during therapy that helped me deal with things and people in a different way” (Participant 6).

In some cases, during the psychotherapy, students discovered they had learning disorders that impaired their academic performance and caused mental distress.

#### Leisure activities

With 33.3% of responses, this category involves students’ experience with leisure activities, such as drawing (5.0%), physical activity (5.0%), and having a pet (5.0%). The narratives show that having a pet was an opportunity to develop affection and reduce loneliness. Drawing helped them believe more in themself and organize their thoughts and physical activity helped to alleviate the stress generated by academic activities and to recover self-esteem.

“Physical activity is important for me. Because I have always liked practicing sports. Then, when I entered college with all the reading and workload, I ended up not doing it anymore. When I started therapy, I started to walk again, to run. And it helped my body a lot. Because I was gaining weight and I was starting to feel bad about my body. And I started doing all the exercises again and I started to feel good again. And I think this is a factor that helped me a lot in the process” (Participant 3).

#### Religious engagement

This category involves the religious experience and its consequences in interpersonal relationships, mentioned by 25% of the students. The students’ narratives mention regularly praying, alone or with a romantic partner, spiritual treatments, and dialogues with participants from the same religious group and their support. For them, prayer was a way of talking to a higher presence and reducing anguish. Religious orientation allowed them to perceive a divine purpose in life, and strength that helped them to hold on during difficult times.

“And religion, I'm a Christian and I'm very devoted to that, to God's purpose, my struggle to get here, to be in this course (...) in this college, it wasn’t in vain, I know it wasn’t, it has to do with God’s purpose. So, I cling to these things, when it's bad, when it's difficult I remember my family, I remember Jesus and everything he did for me and that gives me strength” (Participant 17).

#### Institutional support

Institutional care was mentioned by 20% of the students. They cited the support of professors (15%) and administrative technicians (10%) as important for their recovery because of their understanding and offering to listen and care.

“Then I remember some professors’ understanding. I was taking medication and not able to develop myself into some things, I experienced crises and the professor understood. The reason behind that crisis, because most students did not understand. I had two crises in the classroom, crying crises. And there were people who said that I didn't want to attend class. And I received support from professors that I didn't think would help me” (Participant 9).

#### Strengthening internal resources

This category involved reports of time management skills for class projects or internships and organization of study time (15%). The students mentioned that organizing their home and study routine was important for them, providing a sense of stability. Particularly for those who complained about anxiety disorder, joining a project or an internship helped to slow down thoughts and set short-term goals, reduced the guilt due to not having graduated yet, and increased their chances for employment, as their future was vague and uncertain.

“So I would include my work even though it's a little difficult, and a little stressful. Work was something that helped me focus more, focus on what I wanted to do, what I had to do and what it was, I don't know, I think this changes my thinking sometimes. (...), because when I only studied or stood still - not moving, (...) the thoughts were very fast. (...) Yes, setting short-term goals, the things I needed. I think that for an anxious and shortsighted person like me, spending five years in college, committed only to college is difficult” (Participant 7).

#### Medical treatment

This category was mentioned by 10% of the participants in their responses. The students' narratives indicated that medication helped to reduce the symptoms of sadness, anxiety, and insomnia.

“Over time, I began with psychiatric treatment and started to take medication and then it got better, you know, I started to see some things, I started to get better. I began to see things more clearly about what I was going through, the extent and range of what I was going through” (Participant 19).

#### Complementary treatment

In this category, students mentioned using essential oils (5%), floral remedies (5%), and body therapy (5%) as elements that helped them deal with suffering, reducing sadness and anxiety.

“I won't remember the name, but it's like a home remedy for these anxiety issues, it's like therapy..., floral! I also used it, I believe it helped me a lot because I am very anxious” (Participant 12).

#### Civic engagement

This category concerns participation in social movements, mentioned by 10% of the students. This protective factor was mentioned by those who identified themselves as black and homosexual.

“I started to participate more in social movements. I started to do other things outside my normal routine, things I didn't do during the day, which helped me cope better” (Participant 19).

#### Leaving the course and temporary leave of absence from the university

This category was mentioned by 5% of the students, especially in cases where suicidal ideation evolved into a crisis and required hospitalization.

“Some of it was leaving the environment I was in. So, I set a deadline to leave the university. I stayed away for more or less one period” (Participant 7).

#### Meaning of life

This category was mentioned by 5% of the students and is related to the difficulty of being away from one’s family and home state, existential emptiness, and the need to find meaning regarding the decision to enter university.

“And soon after I began university, I kept looking for that meaning, I didn't have it then. Today I have it and that also makes developing my undergraduate studies and my well-being much easier. Because when you have a sense of direction and you are actually there, it is very important. Right after I began university I didn't see the point. And I wondered why I came here to study, what was the point of being away from family. (...) Why did I look for meaning, why did I come here when in my region there are two private universities where I could be paying or studying through Prouni^1^ or FIES^2^? Today I can clearly say what the reason for all of this was, and I think that if I returned here, I would be very happy” (Participant 1).

#### Interacting with people who have had a mental disorder

This category is related to the experience of interacting with people who have had a mental disorder, which was mentioned by 5% of the students. This experience enabled the stigma associated with the disease to be reduced and enhanced their social support.

“And listening to different people who have been through what I had, and how they helped each other. And I received a lot of advice and it helped me a lot. Based on my own experience, I see many people who have the same problem as mine, but parents and friends, nobody pays attention, and thank God I had a lot of help, and a lot of love” (Participant 16).

### Suicide protective factors as perceived by mental health professionals

The following are the mental health professionals’ most frequent responses: social support (90.9%), psychological treatment (86.4%), leisure activities (81.8%), strengthening internal resources (81.8%), and medical treatment (50.0%). The professionals added a response that was not included in the questionnaire: “complementary therapies (Bach floral remedies and Reiki)”.

### Suicide protective factors perceived by the undergraduate course coordinators

The undergraduate course coordinators produced and presented six categories based on the data analysis and the highest frequency of answers.

#### Employability and opportunities for social ascension offered by the university

This was the category most frequently reported by the undergraduate course coordinators, with 58.3% of responses. When asked about how university life affects the student's mental health, 50.0% of the course coordinators stated that the university course provides the opportunity to expand employment opportunities, enabling better living conditions, while 16.6% stated that the COVID-19 pandemic brought more flexible teaching and facilitated access to knowledge. The narratives show that during the COVID-19 pandemic, technologies were used to facilitate remote teaching activities, offering additional opportunities for students from more distant places. Furthermore, higher education, through the university, offers better job opportunities and, consequently, better living conditions for students, especially for those from underprivileged social classes.

“So, I think this is the role of the university, not only to place excellent professionals on the market but also to potentially change living conditions. If it is providing this, our students who went to Australia or are in Sydney doing a Doctorate, (...) they run after it. Or those who are here or in his Quilombola community,^3^ changing life, or helping to improve the lives of people and friends, as well as their relatives” (Participant 22).

#### Strengthening internal resources

In this category, 50.0% of the course coordinators stated that the university offers the opportunity for a student to learn how to resolve conflicts and grow as a person, and 8.3% mentioned expanding autonomy. The coordinators reported that university life is an opportunity for students to learn how to resolve conflicts. They considered that the situations experienced in the university can prepare a student for future situations in professional life, they represent possibilities for growth and transformation, they can help them make new acquaintances and learn how to take care of their own life. They believed that the pressure experienced during the university routine if appropriately applied by the university, is positive because it helps a student to become an adult and enter the world of work.

“There is a requirement for a change in maturity. (...) The university requires more. So, during a transition phase where the university, their course, is a link to the work environment showing what most of the student's life will be like, they need to incorporate this into the process, let's say, typical of contemporary life, which is work, with very fierce competition in these spaces. Then this real-life process has to begin. Therefore, in the university, these demands are not necessarily bad. We have to find a way to apply it, but here the student or adolescent needs to know, depending on the background they are bringing from school or education, how life will be for this adolescent (...) So I believe that the university provides good challenges in terms of maturing” (Participant 32).

#### Institutional support

In this category, 25% of course coordinators mentioned the support of professors, reporting that students want to be closer to professors, who often provide advice and support.

“A student told me that his family was involved with drugs. (...) Then, when these situations arise, we try to talk. Clearly, offer that friendly shoulder and say “look, let’s move on, come back, don’t lose sight of the objective!”. I think it's direct conversation, this support, with friendly words, I think talking, not leaving them helpless is vital. Help them when they’re at the lowest point” (Participant 22).

#### Social support

In this category, 25% of the course coordinators mentioned the support of classmates as a protective factor, who then became friends and supported each other.

“I have seen that students make many friends during their undergrad years, they have many chances to socialize, I noticed on Instagram that... before the pandemic, you know, they had a group of friends so they could have fun, had a person they could count on. They were always together, in a group. I believe that this social interaction is a support system, it is a positive point that only the university environment offers. You don't find that at work, you probably don't find that in a church, or in any other organization. And at the university, I see that this environment allows them to support each other in order to work on their class project, to be able to study together and solve doubts. During these university years, one has to rely on the other” (Participant 26).

#### Meaning of life

This category had 25.0% of responses, with 16.6% of the undergraduate course coordinators stating that despite the problems experienced by students, the university provides meaning to life and motivation to overcome difficulties. Furthermore, 8.3% stated that the university offers an opportunity for personal fulfillment, especially when a student identifies with the course, or when participating in an internship or job.

“But that's when I say, at the same time that person is going hungry, they’re going through trouble, that person is there every day. They say, “with faith in God I'm going to leave with a diploma” and that person goes on and leaves with a diploma” (Participant 22).

#### University as a refuge from problems

In this category, 8.3% of the course coordinators revealed that the university could be a refuge from problems related to homophobia and other family conflicts.

“Many students occasionally enter university as a refuge from problems they are facing within their families. Sometimes they may leave their hometown, where they live, and go to another municipality. Sometimes the university can help, but at other times, after they arrive at university, this problem gets worse. We've had cases like this, of people who managed to overcome something, sought help from the family, and didn't receive it. (...) Because that student can no longer find support inside their home environment. Their home, family, sexual (orientation) issues, and other issues. And they seek refuge. They have to leave their home because they can no longer live there. So, we have these reports too” (Participant 24).

## Discussion

The objective of this study was to map the protective factors for suicide among college students based on the perception of students, mental health professionals, and undergraduate course coordinators. The findings indicate that social support, strengthening internal resources, institutional support, and finding meaning in the decision to enter university were the protective factors for suicide most reported among the three groups. Although not converging between the three audiences, psychological treatment, leisure activities, religious engagement, medical treatment, civic engagement, employability, opportunities for social ascension offered by the university, and quality family relationships were also frequently cited as protective factors.

The results regarding the centrality of social support as a protective factor for suicide are in line with those found in other studies. Similarly, a study with nationally representative samples shows that individuals with higher social support are 30% less likely to have a suicide attempt during their lifetime, even considering a range of risks and some other protective factors (Kleiman & Liu, 2013). Likewise, a systematic literature review found that in specific subgroups, such as LGBTQIA + young people, support from peers, family members, and teachers is an important protective factor for these adolescents (Luong et al., 2018). A cross-sectional study of Brazilian transgender adults showed similar results (Chinazzo et al., 2021).

More specifically, social support was experienced in different ways: material (help with daily activities), emotional (having someone to share intimate concerns and fears and who gives good advice and information), and affective (demonstrations of affection and having someone to share positive things with); Sherbourne & Stewart, 1991. Support can come from parents, teachers, friends, and intimate partners, who can provide different but complementary support. College students mentioned they received more support from friends than from family, affective partners, or teachers (Tran et al., 2015). These findings are in line with evidence that support from friends is an important suicide protective factor (Nomura et al., 2021; Pereira et al., 2018; Ribeiro & Moreira, 2018), especially for young people from low- and middle-income countries (Aggarwal et al., 2017) and young females, who have greater appreciation of peer support than males (Kerr et al., 2006).

Although reported less frequently than social support from non-professional actors, the students, and mental health professionals asserted that psychological treatment was a relevant protective factor. These findings are not surprising, considering that several studies indicate that psychotherapy and pharmacological treatment reduce the risk of death by suicide (Hazel et al., 2011; Zisook et al., 2012). Furthermore, psychotherapy is central to producing the desired change, presenting a “spillover” effect (Atkins et al., 2017), that is, a positive effect on other related behaviors when the change occurs, such as a decrease in social anxiety and an increase in the person’s socialization, and ultimately an expansion of their social network and an increase in the support received. Additionally, there is evidence that psychotherapy favors self-compassion, a positive and kind attitude towards oneself, identified in a meta-analysis as a protective factor for suicidal behavior (Suh & Jeong, 2021).

However, during the interviews, one student mentioned that he abandoned psychotherapy because he no longer saw the meaning in the process and because the treatment was not in line with his religious principles. Therefore, the effects of psychotherapy should not be overestimated with regard to the way in which it influences the treatment of suicidal ideation and relapse prevention. Factors such as the cultural competence of the therapist must be considered, including their preparation to deal with religious diversity (Davis et al., 2018), so as to avoid students abandoning the treatment.

The mental health professionals attributed greater weight to leisure activities, including engaging in artistic and physical activities and having a pet, as a protective factor than to medical treatment. These findings are aligned with evidence that leisure activities, in addition to reducing the stress generated by academic activities, can help to produce neurotransmitters related to the feeling of well-being, increase self-esteem, facilitate socialization and making new friends, and reduce feelings of loneliness, abandonment, and pathological anxiety (Mushtaq et al., 2014). Likewise, a meta-analysis on cross-sectional studies carried out with adolescents, adults, and older adults found that higher levels of physical activity are associated with lower suicidal ideation (Vancampfort et al., 2018), demonstrating that physical health and mental health are closely associated.

Similarly, students and mental health professionals considered religious engagement to be a relevant protective factor, by offering a sense of hope, as previous studies have shown (Wenjing Li et al., 2020; World Health Organization, 2014). Furthermore, religious beliefs help to reframe traumatic events, adopt new perspectives on life, and discover new meanings for existence. In addition, participating in a religious group increases the chances of acceptance and emotional support, and provides a sense of belonging (Koenig, 2012).

The strengthening of internal resources was mentioned more by the mental health professionals and course coordinators than by the students. Studies indicate that factors such as self-esteem, self-efficacy, social skills (Pereira et al., 2018), self-control, autonomy (Tran et al., 2015) sense of hope (Wenjing Li et al., 2020), and positive development (Aggarwal et al., 2017) are key suicide protective factors for young people. These aspects must be understood considering the young person’s psychosocial development phase since they are in a period of transition from adolescence to adulthood. According to Chickering and Reisser (1993), the development of college students can be understood based on vectors or areas, two of which elucidate the previously mentioned issues. The first consists of managing emotions, in other words, identifying and becoming aware of emotions, and learning to deal with them so as to prevent any harm being caused to daily activities. The second is related to moving towards interdependence, that is, the ability to organize activities and learn to solve problems independently.

Institutional care was also prominent in the findings of this study. This can occur formally (support activities, and inclusion organized by the institution) or informally (the support of teachers and administrative technicians during daily life). Venturini and Goulart (2016) discuss the need to identify the processes that produce exclusion and build a welcoming and inclusive environment in universities. However, previous studies on suicide protective factors have focused strictly on self-esteem, self-efficacy, good family relationships (Pereira et al., 2018), having reasons to live, and a sense of hope (Wenjing Li et al. 2020), while support from professors and university staff has only been briefly addressed in studies (Luong et al., 2018; Marraccini et al., 2022; Tran et al., 2015). The lack of discussions about exclusion and emancipation processes may suggest that the student's suffering and suicidal ideation are interpreted as a psychological and individual issue, therefore leaving its organizational and structural dimensions invisible. Contrary to an individualizing perspective of suicide prevention, institutional support practices guided by a socioecological view, which is inseparable from social justice, can promote the perception of meaning in being at university, employability, and opportunities for professional advancement. These factors are identified as protective for suicide in college students, as evidenced in this study.

Civic engagement, through participation in social movements, mentioned by black homosexual students, is in line with the literature data. A systematic literature review highlighted that LGBTQIA + communities provide emotional support and a space to share experiences and advice for common problems (Luong et al., 2018). Social inequalities and prejudice can often be replicated at university, with detrimental consequences for the student’s mental health, especially for those belonging to minorities (Cornell et al., 2022). Racial/ethnic minority students and first-generation students (students with neither parent having a university degree) often report not fitting in. For example, they sometimes report a perception that they do not belong in the university environment, resulting in negative thoughts that prevent academic and social integration, enhancing depression (Gopalan et al., 2022).

On the other hand, the differences in protective factors identified among the samples should be discussed, allowing some hypotheses to be raised. The first is that mental health professionals may not have answered the questionnaire considering their patient's medical records, but rather based on the information they could retrieve from their memory and theoretical knowledge on the subject. The second hypothesis is that the students, being in a vulnerable position, may have felt uncomfortable in the interviews, and therefore did not share protective factors related to very intimate aspects of their lives, information that the mental health professionals may have had access to and reported in the questionnaire. The third is that the course coordinators gave answers from a long-term perspective, mentioning the influence of the university throughout the training process and after its conclusion, with repercussions for the young person’s personal and professional life. It is assumed that these issues are not perceived by students or reported to mental health professionals, since they are immersed in the educational process, focusing more on specific and short-term situations and, therefore, unable to evaluate the entire process or the gains resulting from the university education.

### Practical implications for suicide prevention

The findings of this study can guide culturally informed interventions for mental health professionals and public administrators. Furthermore, the adoption of a systemic, complex, and integrated approach emphasizes the importance of viewing protective factors as cumulative and interdependent. This necessitates the involvement of multiple informants to gain a deeper and more holistic understanding of the dynamics that protect against suicide among college students. There are five directions for the suicide prevention and health promotion initiatives adopted in universities: encouraging social support in the university community; developing the internal resources of the students; encouraging leisure activities; changing the curriculum structure and professor training to provide meaningful learning that promotes the perception of the meaning of being at the university, and expanding the coverage of the university health services, establishing partnerships with the health network.

After identifying social support, especially from friends, as a suicide protective factor, programs designed to increase peer collaboration should be implemented. College students, student leaders, professors, and administrative staff could be trained to identify and help university students who experience suicidal ideation, a strategy known as gatekeeper (Harrod et al., 2014). Furthermore, other programs that increase the young student’s sense of belonging in the university environment can be developed. It is crucial to create opportunities for college students to get to know each other, make friends, and be able to support each other, especially through healthy interactions between incoming students and seniors.

In addition to intervention in suicidal ideation and crisis, the university should invest in initiatives that anticipate the crisis, aiming to mitigate existing vulnerabilities and strengthen resilience, considering the impact of negative events, as well as develop internal resources (Drum & Denmark, 2012). These initiatives can take place by strengthening the role of student assistance centers in partnership with the course coordinators. They can provide comprehensive psychological support services, essential for nurturing students' well-being and academic success. Universities can also offer interventions to strengthen stress management skills, directed toward understanding developmental transitions and effective ways of managing the stressors experienced. Initiatives of this nature could include discussing the student's life project, and how this project is connected to the academic training, as suggested in Brazilian basic education (Ministério da Educação, 2018).

Leisure Education should be part of wellbeing and promote mental health policy at the university, with consequences for health and wellbeing throughout the life cycle. This term was coined by Stebbins (1999), which integrates the Serious Leisure Perspective (SLP) that divides leisure into three types: serious leisure (activity in which the participant develops a [leisure] career by acquiring and improving skills, knowledge, and experience); casual leisure (relatively short-term pleasant activity that requires little or no special training to enjoy); and project-based leisure (short-term activity carried out in free time that require planning, effort, and sometimes some kind of skill or knowledge, although it is not serious leisure, nor meant to become this) (Stebbins et al., 2007). The three forms of leisure could be discussed and made available at the university, not only for students but for the entire university community. Students can participate in less expensive forms of leisure, such as casual leisure, since other more expensive forms of leisure may not be possible at this moment of life. However, leisure should be viewed more broadly, because it helps to cope with stress and creates a space to interact with others and a channel to express discontent and opposition (Veal, 2017), which can be particularly important for young people. Furthermore, promoting a leisure culture in young people is vital for successful aging throughout adult life (Paggi et al., 2016).

Similarly, actions such as a focus on the mental health of college students at the institutional level are imperative. It is necessary to build social marketing campaigns with students, motivating them by showing opportunities in the job market offered through university education. Actions that review the curriculum structure are equally essential, providing teaching based on competencies and encouraging problem-solving, critical thinking, cooperation, adaptability, coordination, communication, and leadership. The study by Prada et al., (2022) shows that these skills help to improve academic performance, reducing emotional distress and consequently increasing the chances of employability in the future.

In addition, actions that articulate multiple sectors (e.g.: academic performance, social assistance, health, and human rights) are indispensable in order to expand institutional support, mainly by monitoring students in critical phases of the course, such as admission and completion, and strengthening relationships between students and professors. The university should be a space for young people to connect with supportive adults, an environment that fosters feelings of belonging through the enthusiasm and confidence of its actors in the students’ academic performance (Marraccini et al., 2022), especially for those from racial and gender minorities.

The behavior of seeking professional help should be encouraged since psychotherapy and medical treatment have proved to be a relevant protective factor. Therefore, the Psychosocial Care Network and the Psychosocial Care Centers, an integral part of the Brazilian National Health System, must be expanded, making mental health services and multidisciplinary care accessible to all who need them. It is critical to create partnerships with the public health network, aiming to propose agreements that benefit the university and the regional services. The knowledge produced at the university can be offered in the form of courses for professionals in the region, qualifying the service offered to the population, and, more specifically, to college students. On the other hand, the services in the area, including those arising from public health, education, and social assistance policies, could increase assistance to students and their families.

Partnerships with the public health network could also contribute to improving epidemiological data production. A systematic literature review emphasized that in low- and middle-income countries there is a lack of reliable epidemiological data on attempted and completed suicides, in addition to low data availability on risk and protective factors for suicide among college students (Breet et al., 2021). This scenario contributes to the fact that suicide is not considered to be a serious public health problem, limiting efforts for its prevention.

### Limitations

The study has some limitations. Considering that approximately one-third of individuals with suicidal ideation attempt suicide (Christensen et al., 2021; Nock, et al., 2008), caution should be applied when generalizing the findings about the protective factors for suicidal ideation and those for real attempts. Studies with larger samples could investigate whether protective factors are different for college students with suicidal ideation and for those who have already attempted suicide and whether protective factors vary according to gender, ethnicity, and sexual orientation. Two considerations are required when interpreting the results. First, the sample included students from a public university in the North of Brazil. The results may not apply to young people from other geographic groups or who are not in higher education. Second, data collection was conducted through videoconference interviews, and this may have contributed to the students and/or course coordinators not feeling comfortable about revealing certain issues.

## Conclusion

The findings can guide mental health policies for young people, especially in higher education institutions. Although the role of the university is professional training, understanding which lines sustain the young person’s life and how they are interconnected makes training a process that not only transmits knowledge but also contributes to human development. Furthermore, it is crucial to go beyond the clinical paradigm and understand that suicide prevention is not only restricted to offering psychotherapy and drug treatment but also entails actions to promote the health of college students with a view to expanding the social support network, strengthening personal resources (such as autonomy and self-efficacy), improving institutional support, increasing the perception of belonging and promoting the practice of leisure activities.

Data can support research plans in many directions. More studies on protective factors for suicide in college students are needed, especially those with a broader audience, separating data from students with suicidal ideation from those who have attempted suicide. Future studies should investigate protective factors, as well as suicidal thoughts and behaviors, both longitudinally and experimentally, to examine whether interventions that reinforce protective factors can reduce the risk of suicide over time. In addition to the academic community and mental health professionals, new studies could include parents as participants. The data derived from these studies could guide the preparation of the theory of change of the programs for suicide prevention in college students, as well as guide the development, implementation, and evaluation of these programs.

## Data Availability

The datasets used and/or analyzed during the current study are available from the corresponding author upon reasonable request.

## Abbreviations

CNS: Conselho Nacional de Saúde (National Health Council of Brazil)
FIES: Fundo de Financiamento Estudantil (Student Financing Fund of Brazil)
PROUNI: Programa Universidade Para Todos (University for All Program of Brazil)

## Footnotes

1 The “Programa Universidade para Todos” (PROUNI) is a Brazilian government initiative that provides scholarships to underprivileged students for higher education in private universities.

2 The “Fundo de Financiamento Estudantil” (FIES) is a Brazilian government program that offers loans to students for covering tuition fees at private higher education institutions.

3 A Quilombola community is a village formed by people descended from black enslaved people in Brazil.
